# Identification of Atypical Enteropathogenic *Escherichia coli* O98 from Golden Snub-Nosed Monkeys with Diarrhea in China

**DOI:** 10.3389/fvets.2017.00217

**Published:** 2017-12-13

**Authors:** Mingpu Qi, Qiankun Wang, Shengtao Tong, Gang Zhao, Changmin Hu, Yingyu Chen, Xiang Li, Wanji Yang, Yuchen Zhao, Sara Platto, Robertson Ian Duncan, Jianguo Chen, Huanchun Chen, Aizhen Guo

**Affiliations:** ^1^State Key Laboratory of Agricultural Microbiology, Wuhan, China; ^2^College of Veterinary Medicine, Wuhan, China; ^3^College of Animal Science, Wuhan, China; ^4^Hubei Conservation and Research Center for the Golden Monkey, Shennongjia, China; ^5^Hubei Province Key Laboratory of Conservation Biology of Shennongjia Golden Monkey, Shennongjia, China; ^6^China-Australia International Joint Research and Training Centre for Veterinary Epidemiology, Huazhong Agricultural University, Wuhan, China

**Keywords:** enteropathogenic *Escherichia coli*, *Rhinopithecus roxellana*, diarrhea, virulence, non-human primates

## Abstract

Fecal samples (*n* = 76) were collected from 38 snub-nosed monkeys (*Rhinopithecus roxellana*) in Shennongjia National Nature Reserve (China) and examined for the presence of enteropathogenic *Escherichia coli* (EPEC). The 56 samples originated from 30 free-ranging monkeys on the reserve and 20 samples from 8 captive monkeys that were previously rescued and kept at the research center. Eight diarrhea samples were collected from four of the eight captive monkeys (two samples from each monkey), and two EPEC strains (2.6%) (95% confidence interval 0.3–9.2%) were isolated from two fecal samples from two diarrheic monkeys. Both strains belonged to serotype O98 and phylogenetic group D (*TspE4C2*^+^, *ChuA*^+^). The virulence gene detection identified these strains as an atypical EPEC (aEPEC) (*bfpB^**–**^, stx1^**–**^*, and *stx2*^–^) with the subtype *eae*^+^, *escV*^+^, and *intimin*β^+^. These strains were highly sensitive to all the antibiotics tested. The lethal dose 50% of the two isolates in Kunming mice was 7.40 × 10^8^ CFU/0.2 mL and 2.40 × 10^8^ CFU/0.2 mL, respectively, indicating low virulence. Based on the report that this serotype had been isolated from some other non-human animals and humans with diarrhea, the first identification of aEPEC O98 strains and their drug resistance profile in *R. roxellana* is of ecological significance for disease control in this endangered species.

## Introduction

The golden snub-nosed monkey (*Rhinopithecus roxellana*) is a listed class A protected and endangered (EN) species in the International Union for Conservation of Nature (IUCN) Red List.^1^ It is distributed in the Sichuan, Gansu, Shanxi, and Hubei provinces in China (1, 2). Diseases represent one of the major threats to the survival of these monkeys (1), and therefore, it is important to identify pathogens of these animals, which may affect their health. However, there has been little research published on potential pathogens of golden snub-nosed monkeys (3–5). The main obstacles to conducting research in this species are the difficulties in accessing the monkeys and in collecting suitable samples in a non-invasive way.

Feces is the most readily available type of sample for many animals. Enteropathogenic *Escherichia coli* (EPEC) is a major pathogen identified in feces that causes infantile diarrhea in humans in both developing and developed countries and results in thousands of deaths worldwide each year (6, 7). The pathogenesis of EPEC depends on the chromosomal pathogenicity island locus of enterocyte effacement (LEE). The LEE contains a number of genes, including *eae*, which is essential for inducing the formation of characteristic attaching and effacing lesions in the intestinal epithelium (8). EPEC strains are classified as typical EPEC and atypical EPEC (aEPEC) on the basis of the presence of *E. coli* adherence factor plasmid, which carries the *bfp* gene, encoding the bundle-forming pili. Typical EPEC strains have mainly been isolated from humans, but they have been identified in other animals such as monkeys, dogs, cats, birds, and deer (9–12). They are considered to be a leading cause of infantile diarrhea in developing countries (13). The most important epidemiological feature of EPEC infection is its significantly high prevalence in children aged 0–5 years (14, 15).

In addition, aEPEC strains have been isolated from a wide range of hosts including calves, dogs, cats, cervids, sheep, goats, pigs, non-human primates, and humans (9, 16–21). In addition, the aEPEC strains isolated from non-human primates and humans have a very high degree of similarity (9, 22), underlying their zoonotic nature.

The purpose of this study was to identify EPEC strains in the feces of golden snub-nosed monkeys in the Shennongjia National Nature Reserve, PR China, and to provide information to aid in disease control and protection of this EN species.

## Materials and Methods

### Ethics Statement

This study was carried out in accordance with the recommendations of Hubei Regulations for the Administration of Affairs Concerning Experimental Animals (2005), Ethical Committee for Experimental Animals of Huazhong Agricultural University. The protocol was approved by the Ethical Committee for Experimental Animals of Huazhong Agricultural University (Permit Number: SYX–K(ER)2010-0029).

### Sample Collection

**Figure 1:**
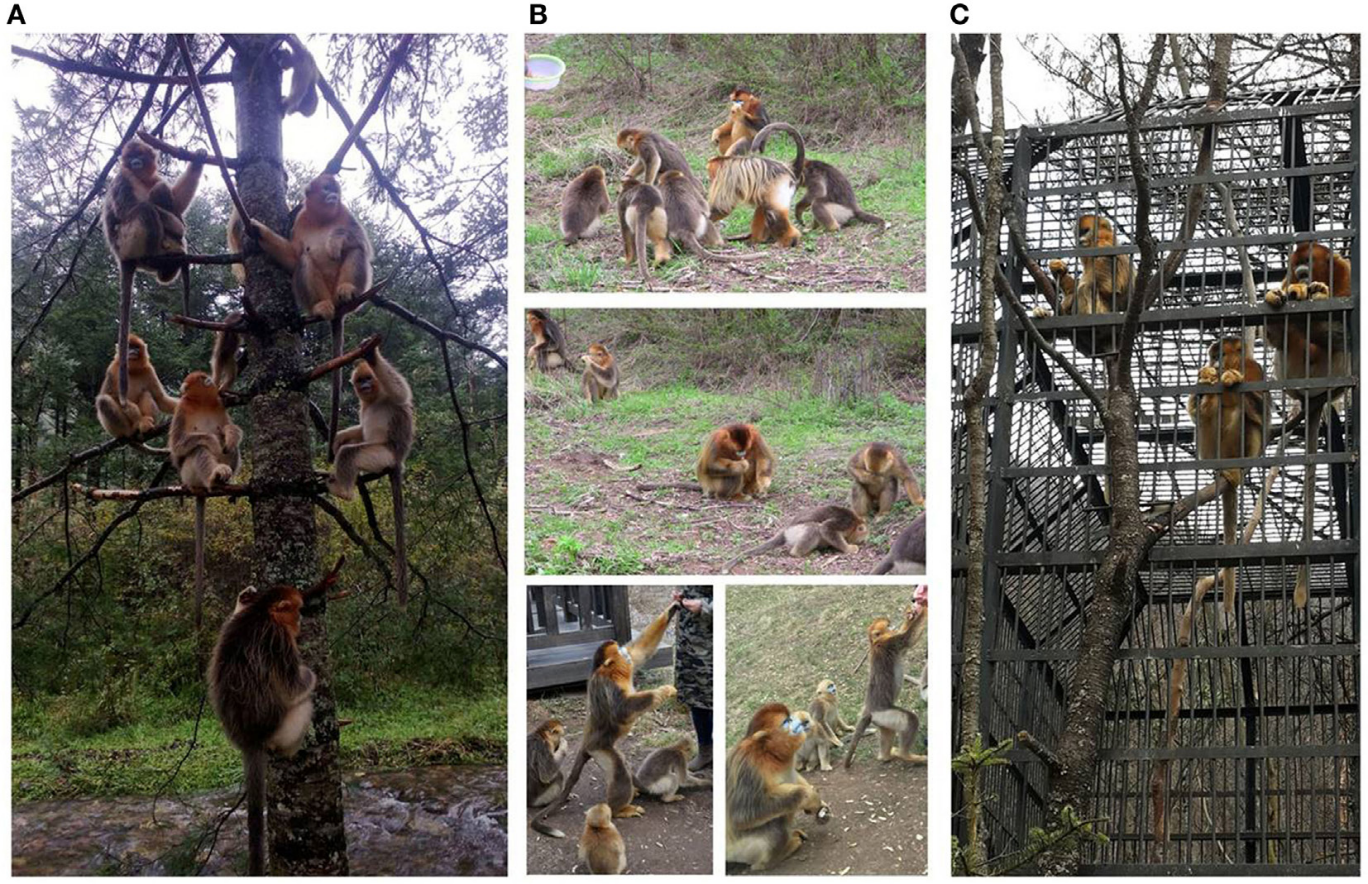
Monkeys sampled in this study. **(A)** The monkeys from a one-male and multi-female unit waiting for food in the tree before feeding time; **(B)** monkeys fed with different food, which varies with the seasons; **(C)** the monkeys kept in the cage which is located in an open area.

The Shennongjia National Nature Reserve hosts approximate 1,280 monkeys distributed in 8 areas, with 80 wild monkeys in Dalongtan area (Figure 1A) and 8 captive monkeys in Xiaolongtan area. The accessible monkeys included a community of 30 free-ranging monkeys in Dalongtan and 8 captive monkeys in Xiaolongtan. These two areas are connected with the valley in the Hubei Conservation and Research Center for the Golden Monkey at Shennongjia National Nature Reserve, Hubei Province, China.

The community was established at Dalongtan area in 2006 and is composed of five families: four one-male and multi-female units and one all-male unit. The monkeys gather at a feeding station (Figure 1A) three times a day (10:00–11:00, 14:00–15:00, and 18:00–19:00 h) where one of the staff from the observation station feeds them with sweet potatoes, oranges, peaches, apples, or peanuts. The food provided by the observation station to the monkeys varies with the season (Figure 1B). During the intervals between feeding periods, the monkeys return to the forest where they eat forage like other wild monkeys. The eight captive monkeys (BB2, QQ1, QQ2, HH3, YY2, TT, JJ, and LL2) kept at Xiaolongtan (Figure 1C) were rescued from different areas of Shennongjia Reserve (Table 1). These animals had physical injuries caused by fighting in the adults (BB2, QQ1, QQ2, HH3, YY2, and TT) and by falling from tree branches in the sub-adults (JJ and LL2) when they were saved. After recovery, they were kept at Xiaolongtan for research. Their enclosures are located in an open place (Figure 1C); the floors of the enclosures are washed daily with running water from a hose by the staff of the center. The water from the floor cleaning disperses into the surrounding environment.

**Table 1:** Information on golden snub-nosed monkeys with fecal samples.

Sampling time	Location	Monkey ID	Sex	Age	Health status	Number of samples collected
July–September 2013	Dalongtan	BT	M	Adult	H	2
		DD1	F	Adult	H	2
		HH1	F	Adult	H	2
		YY1	F	Adult	H	2
		TJ	F	Sub-adult	H	1
		DWB	F	Adult	H	2
		NN	M	Adult	H	2
		XB1	F	Adult	H	2
		XH	F	Adult	H	2
		HH2	F	Adult	H	2
		ME	F	Sub-adult	H	2
		XY	F	Sub-adult	H	2
		XB2	M	Adult	H	2
		LN	F	Adult	H	2
		GG	F	Adult	H	2
		FF	M	Sub-adult	H	1
		YZ	F	Sub-adult	H	1
		YE	F	Sub-adult	H	1
		XXL	M	Sub-adult	H	1
		YJ	M	Sub-adult	H	2

	Xiaolongtan	BB2	M	Adult	D	2
		QQ1	F	Adult	H	3
		QQ2	M	Adult	H	3
		HH3	F	Adult	D	2
		YY2	F	Adult	D	2
		TT	M	Adult	H	3
		JJ	F	Sub-adult	D	2
		LL2	F	Sub-adult	H	3

March 2014	Dalongtan	XX	M	Adult	H	2
		BB1	F	Adult	H	2
		HHE	F	Adult	H	2
		LL1	F	Adult	H	2
		SB	F	Sub-adult	H	2
		DW	M	Sub-adult	H	2
		XHT	M	Adult	H	3
		WY	M	Adult	H	2
		YH	M	Adult	H	2
		JW	M	Sub-adult	H	2

Total						76

*adult, more than 5 years; D, diarrhea; H, healthy; sub-adult, 2–4 years*.

Each monkey was individually identified by giving a unique name and code based on its hair color, body shape, and other morphological features. Fecal samples were collected during three field trips of 1 week’s duration each in August and October 2013 and in March 2014.

Each time, the samples were collected by two researchers who were familiar with the monkeys before the morning (10:00–11:00 h) and afternoon (14:00–15:00 h) feeding periods, when the monkeys were waiting for their food (Figure 1A). Each researcher was responsible for one family at a time. As soon as the monkeys defecated, the feces were collected immediately. The fresh feces were immediately placed in sterile disposable plastic bags and stored in a portable refrigerator at 2°C–8°C. Within 2 h after collection, the samples were transported to the laboratory and kept at −20°C until further analysis.

### Detection of Fecal Pathogens

The fecal samples were sent to the Testing Department, XISHAN Biotechnology Inc. (Vanton Research Laboratory, Suzhou, China) for the detection of common bacterial pathogens (Catalogue# PA21) including *E. coli, Salmonella*, and *Shigella*, following the protocol from China national standard GB/T14926.11-2001 (23), GB/T 14926.1-2001 (24), GB/T 14926.47-2008 (25), and for *Campylobacter* spp. and *Yersinia* with real-time PCR (qPCR).

For *E. coli* isolation, fecal samples were inoculated on MacConkey agar plates (Oxoid, England) and incubated at 37°C for 16–24 h (23). The morphological and cultural characteristics and biochemical properties were analyzed according to previous protocols (26).

For *Salmonella* isolation, the fecal samples were cultured in selenite enrichment broth at 37°C overnight, and thereafter the culture was transferred on *Shigella* and *Salmonella* agar plates for 18–24 h at 37°C (24). The suspicious colonies were subjected to PCR amplification with primers specific to the *ITS* gene (Table 2).

**Table 2:** Primer sequences, annealing temperatures, and sizes of amplified fragments from selected genes of the target pathogens.

Primers	Sequence(5′–3′)	Product size (bp)	Anneal temperature (°C)	Pathovar	Reference
*TspE4C2-F*	GAGTAATGTCGGGGCATTCA	152	55	*E*. *coli* phylogenetic groups	Clermont et al. (27)
*TspE4C2-R*	CGCGCCAACAAAGTATTACG				
*ChuA-F*	GACGAACCAACGGTCAGGAT	279	55		Clermont et al. (27)
*ChuA-R*	TGCCGCCAGTACCAAAGACA				
*YjaA-F*	TGAAGTGTCAGGAGACGCTG	211	55		Clermont et al. (27)
*YjaA-R*	ATGGAGAATGCGTTCCTCAAC				
*eae-F*	TCAATGCAGTTCCGTTATCAGTT	482	58		Vidal et al. (28)
*eae-R*	GTAAAGTCCGTTACCCCAACCTG				
*escV-F*	ATTCTGGCTCTCTTCTTCTTTATGGCTG	544	62		Antikainen et al. (29)
*escV-R*	CGTCCCCTTTTACAAACTTCATCGC				
*bfpB-F*	GACACCTCATTGCTGAAGTCG	910	60		Antikainen et al. (29)
*bfpB-R*	CCAGAACACCTCCGTTATGC				
*Intimi*α	CCTTAGGTAAGTTAAGT	558	52	EPEC	Adu-Bobie et al. (30)
*Intimi*β	TAAGGATTTTGGGACCC	562	50		
*Intimi*γ	ACAAACTTTGGGATGTTC	562	58		
*Intimi*δ	TACGGATTTTGGGGCAT	563	52		
*Reverse*	TTTATGTGCAGCCCCCCAT				
*Intimi*ε*-F*	CCCGAATTCGGCACAAGCATAAGC	2,608	68		Oswald et al. (31)
*Intimi* ε*-R*	AGCTCACTCGTAGATGACGGCAAGCG				
*stx1A-F*	CGATGTTACGGTTTGTTACTGTGACAGC	244	62		Müller et al. (32)
*stx1A-R*	AATGCCACGCTTCCCAGAATTG			EHEC	
*stx2A-F*	GTTTTGACCATCTTCGTCTGATTATTGAG	324	61		Müller et al. (32)
*stx2A-R*	AGCGTAAGGCTTCTGCTGTGAC				
*elt-F*	GAACAGGAGGTTTCTGCGTTAGGTG	655	60		Müller et al. (32)
*elt-R*	CTTTCAATGGCTTTTTTTTGGGAGTC				
*estIa-F*	CCTCTTTTAGYCAGACARCTGAATCASTTG	157	62	ETEC	Müller et al. (32)
*estIa-R*	CAGGCAGGATTACAACAAAGTTCACAG				
*estIb-F*	TGTCTTTTTCACCTTTCGCTC	171	58		Müller et al. (32)
*estIb-R*	CGGTACAAGCAGGATTACAACAC				
*invE-F*	CGATCAAGAATCCCTAACAGAAGAATCAC	766	62		Müller et al. (32)
*invE-R*	CGATAGATGGCGAGAAATTATATCCCG			EIEC	
*astA-F*	TGCCATCAACACAGTATATCCG	102	58		Antikainen et al. (29)
*astA-R*	ACGGCTTTGTAGTCCTTCCAT				
*aggR-F*	ACGCAGAGTTGCCTGATAAAG	400	58	EAEC	Antikainen et al. (29)
*aggR-R*	AATACAGAATCGTCAGCATCAGC				
*pic-F*	AAATGTCAGTGAACCGACGATTGG	1,111	60		Antikainen et al. (29)
*pic-R*	AGCCGTTTCCGCAGAAGCC				
*ITS-F*	TATGCCCCATCGTGTAGTCAGAAC	312	58	*Salmonella* spp.	Park et al. (33)
*ITS-R*	TGCGGCTGGATCACCTCCTT				
*virF-F*	AGCTCAGGCAATGAAACTTTGAC	618	58	*Shigella* spp.	Vidal et al. (28)
*virF-F*	TGGGCTTGATATTCCGATAAGTC				
*IpaH-F*	CTCGGCACGTTTTAATAGTCTGG	933	58	*Shigella* spp.	Vidal et al. (28)
*IpaH-R*	GTGGAGAGCTGAAGTTTCTCTGC				

*EAEC, enteroaggregative Escherichia coli; EHEC, enterohemorrhagic Escherichia coli; EIEC, enteroinvasive Escherichia coli; EPEC, enteropathogenic Escherichia coli; ETEC, enterotoxigenic Escherichia coli*.

For *Shigella* isolation, the fecal samples were cultured on MacConkey agar plates at 37°C overnight (25), following which suspicious colonies were subjected to PCR amplification with primers specific to the *virF* and *ipaH* genes (Table 2).

For *Campylobacter* spp. and *Yersinia* detection, the fecal samples were mixed with phosphate-buffered saline and centrifuged at 500 *g* for 10 min, and the supernatant was used to extract the DNA using a TianGen DNA extraction kit (TianGen, Beijing, China) according to the manufacturer’s instructions. The DNA was subjected to qPCR with the primers and reaction conditions described in previous reports (34, 35).

In addition, the presence of diarrhea-related parasitic helminths (Catalogue# PA31) was investigated by microscopic observation of worm eggs, following the protocol from China national standard GB/T 18448.6-2001 (36). Briefly, fecal samples were mixed with saturated saline in a 5-mL tube and allowed to rest for 5 min. After discarding large floating objects, the tubes were filled with saturated saline. A cover glass was pressed gently on top of the liquid level and allowed to sit for 20 min, and the sample was then observed under a microscope for worm egg examination.

### PCR-Based Genotyping of *E. coli* Isolates

Genotyping of *E. coli* was performed using a triplex PCR assay with a combination of three gene markers (*ChuA, yjaA*, and *tspE4C2*) (27, 37). The resulting PCR products allowed classification of the strains into one of the four major phylogenetic lineages: A1, B1, B2, and D. The primer sequences for the PCR and the sizes of the amplified products are listed in Table 2. The primers were synthesized by Sangon Biological Engineering Technology and Service Co., Ltd. (Shanghai, China).

Each PCR was performed in a final volume of 25 µL, which included 12.5 µL of 2× PCR Mix (TransGen, Beijing, China), 0.5 µL of 10 nmoL of each primer, 11 µL of ddH_2_O, and 1 µL of bacterial culture. All PCRs were performed with an initial denaturation step at 94°C for 5 min followed by 30 cycles of denaturation at 94°C for 30 s, annealing at 55°C for 30 s, extension at 72°C for 30 s, and a final single extension step at 72°C for 5 min. PCR products were detected with electrophoresis in 1% agarose gels, along with a DL2000 DNA ladder (TaKaRa, Dalian, China), and visualized under UV illumination after staining with ethidium bromide.

### Analysis of Virulence Factors with PCR

Diarrheagenic *E. coli* falls into six categories based on the virulence markers: enteropathogenic *E. coli* (EPEC), enterotoxigenic *E. coli* (ETEC), enterohemorrhagic *E. coli* (EHEC), enteroinvasive *E. coli* (EIEC), enteroaggregative *E. coli* (EAEC), and diffuse-adhering *E. coli* (28, 29, 32, 38). Twelve virulence-associated genes (*eae, escV, bfpB, stx1A, stx2A, elt, estIa, estIb, invE, astA, aggR*, and *pic*) were selected to differentiate these categories of diarrheagenic *E. coli* with PCR, with the specific primers listed in Table 2. The *eae*-positive *E. coli* isolates were analyzed further to determine the *eae* subtypes of α, β, γ, δ, and ε (30, 31). All the primers were synthesized by Sangon Biological Engineering Technology and Service Co., Ltd. (Shanghai, China).

### Serotyping of Isolates

Serotyping was performed by employing a standard slide agglutination test with standard antisera purchased from the China Institute of Veterinary Drug Control (26, 39). The *E. coli* strains were grown overnight in tryptic soy broth (BD, New Jersey, USA) and then autoclaved at 1.05 kgf/cm^2^ for 2 h. The strains were serotyped with standard antisera against all antigens of EPEC, ETEC, EHEC, STEC, EAEC, and EIEC of O1–O163, according to the manufacturer’s instructions.

### Lethal Dose 50% (LD_50_) Test of the Isolates

The LD_50_ in mice was determined using a protocol described previously (40, 41). Briefly, female-specific pathogen free Kunming mice aged 4–6 weeks were purchased from China Hubei Provincial Center for Disease Control and Prevention. Each group included six mice that were injected intraperitoneally with the bacterial culture. The LD_50_ dose was calculated on the basis of Karber’s formula (40).

### Antimicrobial Sensitivity Testing

Bacterial antimicrobial susceptibility was determined using VITEK 2 (BioMérieux, Hazelwood, MO, USA) for all 16 drugs in 6 different categories: aminoglycosides: amikacin, gentamicin, and tobramycin; cephalosporins: cefepime, cefotetan, ceftazidime, ceftriaxone, and cephazolin; β-lactam: ampicillin, aztreonam, and meropenem; fluoroquinolones: ciprofloxacin and levofloxacin; penicillins: imipenem and piperacillin; sulfonamide: cotrimoxazol. Each antibiotic was twofold serially diluted from 128 to 0.125 µg/mL. *E. coli* strain ATCC 25922 was used as the quality control strain. The final results were interpreted as sensitive (S), intermediate (I), or resistant (R) on the basis of the Clinical and Laboratory Standards Institute (42) Guidelines.

### Statistical Analysis

The positive rate (%) of the samples for pathogen isolation was defined as number of positive specimens/number of specimens tested. The 95% confidence interval (CI) for the positive rate was calculated with online epitools, which were based on the method described previously (43).^2^

## Results

### Sample Collection

A total of 76 fecal samples were collected, which included 56 samples from 30 free-ranging monkeys in the Dalongtan area and 20 samples from 8 captive monkeys in the Xiaolongtan area, with an average of 1–3 samples from each monkey. Among all the samples collected, eight were diarrheic feces from four captive monkeys with diarrhea, whereas all the other fecal samples were normal in shape, dark, and odorless (Table 1).

### Pathogen Detection

Two *E. coli* strains were isolated from the 76 samples, with a positive rate in feces of 2.6% (2/76) (95% CI, 0.3–9.2%). The morphological and cultural characteristics and biochemical properties of the isolates determined them to be typical *E. coli*. In addition, both strains were from two of the four captive monkeys with diarrhea, who were kept in different enclosures. The positive rate of the strains in the diarrheic monkeys was 50.0% (2/4) (95% CI, 6.8–93.2%), while a positive rate of 0% (0/34) (95% CI, 0.0%, 10.3%) was found in the other 34 monkeys without diarrhea.

The morphological and cultural characteristics and biochemical properties were in agreement with the standards of typical *E. coli*. The isolates produced smooth, circular and bright pink or red colonies on MacConkey agar plates. The culture smear was identified as Gram negative by observation under the light microscope. Biochemical tests demonstrated that the isolates could ferment glucose, sorbitol, and xylose, and they were positive in the indole and methyl red tests. Moreover, the isolates could utilize ornithine and lysine, but they could not produce hydrogen sulfide and were negative in the Voges–Proskauer reaction.

In addition, all fecal samples were negative for other pathogens commonly associated with diarrhea such as *Salmonella, Shigella, Campylobacter, Yersinia*, and helminths.

### Genotyping and Serotyping of *E. coli* Isolates

**Figure 2:**
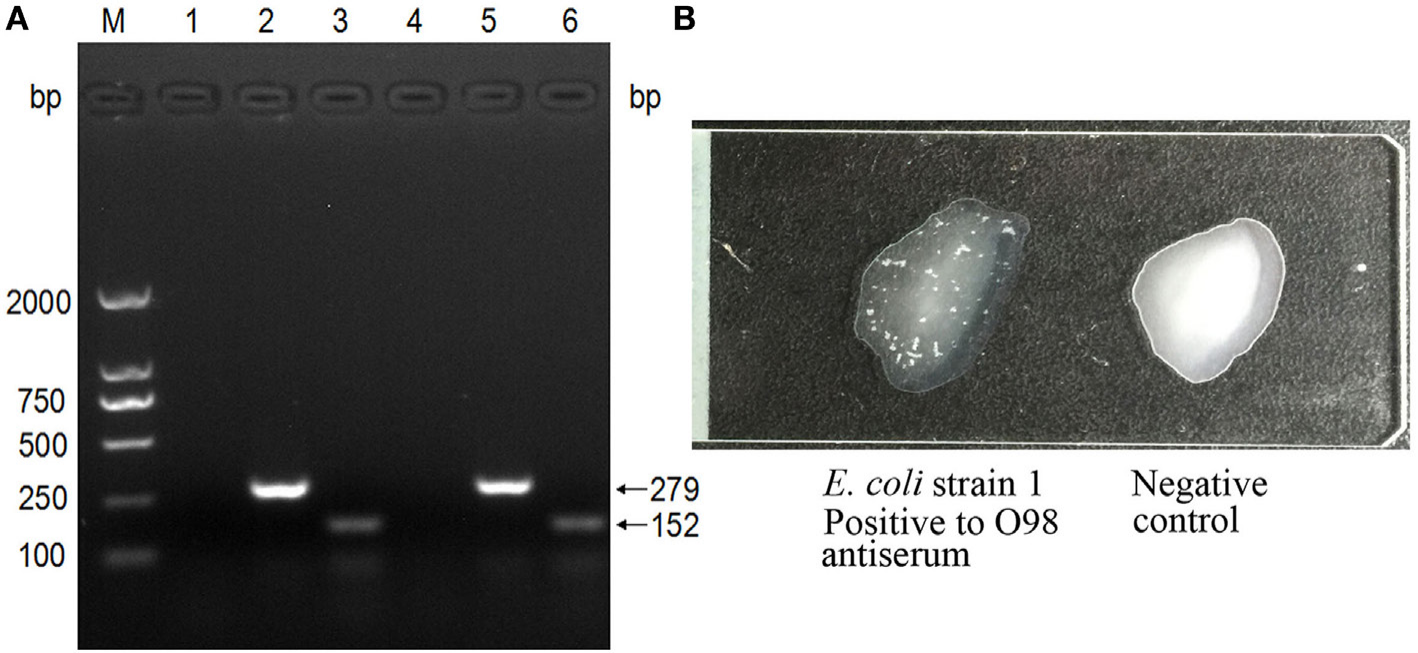
Genotyping and serotyping of *Escherichia coli* isolates. **(A)** The triplex PCR specific for *E. coli* phylogenetic groups. (M) DNA ladder (DL2000); lanes 1–3: PCR products with primers specific to the genes *yjaA, ChuA*, and *TspEC2*, using the template of strain 1; lanes 4–6: PCR products with primers specific to the genes *yjaA, ChuA*, and *TspEC2*, using the template of strain 2. **(B)** The strains were typed by the slide agglutination test with standard antisera against all antigens of enteropathogenic *E. coli* (EPEC), enterotoxigenic *E. coli* (ETEC), enterohemorrhagic *E. coli* (EHEC), enteroinvasive *E. coli* (EIEC), enteroaggregative *E. coli* (EAEC), and diffuse-adhering *E. coli* (DAEC) of O1–O163. The left was O98 positive, shown by the apparent white agglutinating clusters, while the right was negative.

The PCR-based genotyping was performed with a triplex PCR. The *TspE4C2* (152 bp) and *ChuA* (279 bp) amplicons were found to co-exist, confirming that both strains belong to group D (Figure 2A).

**Figure 3:**
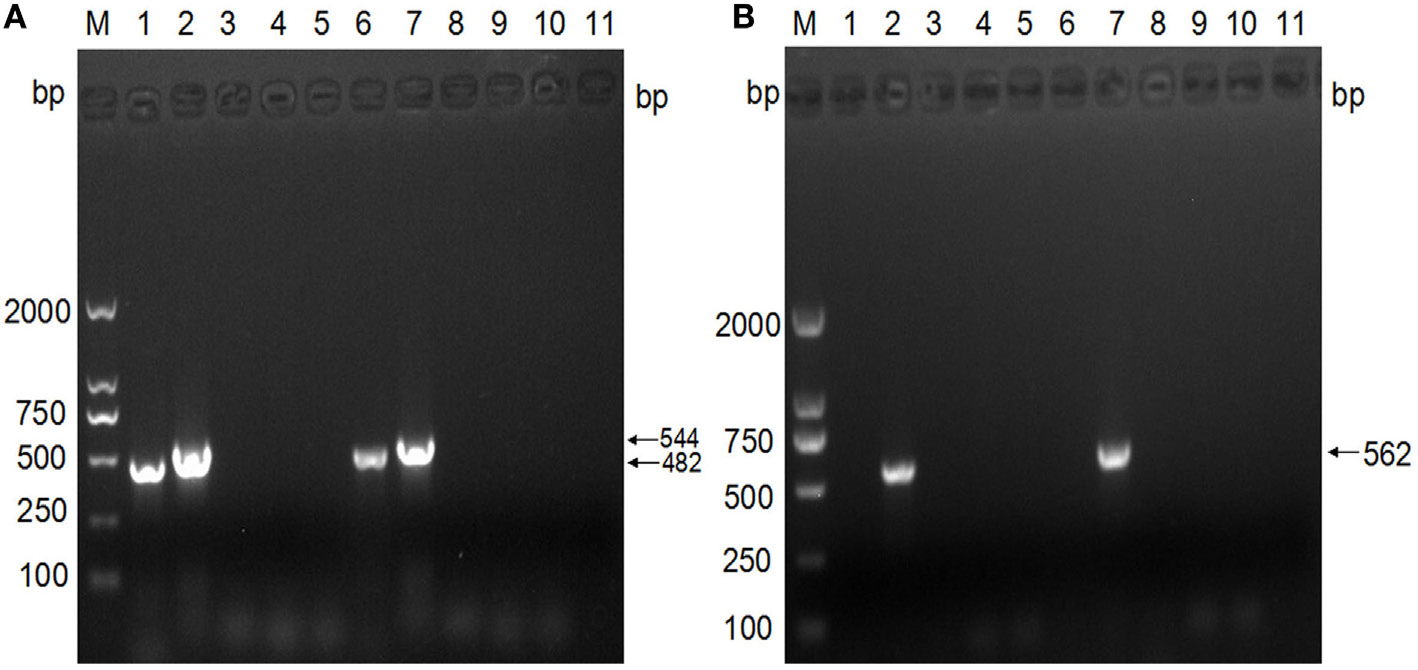
Subgenotyping of *Escherichia coli* isolates based on the virulence genes. **(A)** PCR analyses of virulence factors. M: DNA ladder (DL2000); lanes 1–5: PCR products with primers specific to the genes *eae, escV, bfpB, stx1*, and *stx2*, using the template of strain 1; lanes 6–10: PCR products with primers specific to the genes *eae, escV, bfpB, stx1*, and *stx2*, using the template of strain 2; lane 11: negative. **(B)** PCR analysis of intimin subtypes. M: DNA ladder (DL2000); lanes 1–5: PCR products with primers specific to *intimin* α, *intimin* β, *intimin* γ, *intimin* δ, and *intimin* ε, using the template of strain 1; lanes 6–10: PCR products with primers specific to *intimin* α, *intimin* β, *intimin* γ, *intimin* δ, and *intimin* ε, using the template of strain 2; lane 11: negative control.

The analysis of the virulence genes showed that both strains possess the *eae* (482 bp) and *escV* (544 bp) gene amplicons, but lacked *bfpB, stx1*, and *stx2*. These findings confirmed that the isolates are aEPEC strains (Figure 3A). Further subtyping of the virulence genes with regard to the *eae* type showed the presence of only the amplicon of the *intimin* β gene (562 bp), which indicates that both strains belong to the β subtype (Figure 3B).

In addition, serotyping with the standard antiserum to O antigens demonstrated that both strains are of serotype O98 (Figure 2B).

### LD_50_ Testing of *E. coli* Isolates

The LD_50_ of the two *E. coli* strains was tested in mice to determine their virulence. The mice were injected intraperitoneally with the bacteria and observed for 36 h. The mice who challenged with the EPEC strains began to die at 24 h post-challenge and the mortality being positively correlated to the bacterial concentrations in the next 12 h, while the mice in the control group were healthy. The strains had LD_50_ of 7.40 × 10^8^ CFU/0.2 mL and 2.40 × 10^8^ CFU/0.2 mL, respectively (Table S1 in Supplementary Material), which demonstrates that they belong to the class of low virulence.

### Antimicrobial Susceptibility of *E. coli* Isolates

*Escherichia coli* strain ATCC 25922 and the two isolates were cultured overnight, and then 0.1 mL fresh culture of each strain was streaked on Luria-Bertani agar plates and incubated for 12 h, and the antimicrobial susceptibility of each strain was tested by VITEK 2. As expected, the quality control strain was sensitive to all 16 antibiotics that were tested. Meanwhile, both aEPEC isolates were discovered to be similarly sensitive to all 16 antibiotics (Table 3).

**Table 3:** Antibiotic susceptibility testing of atypical EPEC isolates.

Category	Antibiotics (128 µg/mL)	MIC (μg/mL)/interpretation	
		Strain 1	Strain 2
Aminoglycosides	Amikacin	≤2/S	≤2/S
	Gentamicin	≤1/S	≤1/S
	Tobramycin	≤1/S	≤1/S

Cephalosporins	Cefepime	≤1/S	≤1/S
	Cefotetan	≤4/S	≤4/S
	Ceftazidime	≤1/S	≤1/S
	Ceftriaxone	≤1/S	≤1/S
	Cephazolin	≤2/S	≤2/S

β-lactams	Ampicillin	≤2/S	≤2/S
	Aztreonam	≤1/S	≤1/S
	Meropenem	≤1/S	≤1/S

Fluoroquinolones	Ciprofloxacin	≤0.25/S	≤0.25/S
	Levofloxacin	≤0.25/S	≤0.25/S

Penicillins	Imipenem	≤1/S	≤1/S
	Piperacillin	≤4/S	≤4/S

Sulfonamide	Cotrimoxazol	≤20/S	≤20/S

*EPEC, enteropathogenic Escherichia coli; S, sensitive*.

## Discussion

The aEPEC is a well-known zoonotic pathogen that causes diarrhea in a wide range of hosts including humans and different species of non-human animals (9, 16–21). In this study, both strains were identified as group D serotype O98. This type of aEPEC serotype is not among those usually affecting humans, as defined by the World Health Organization (44), which include O26, O55, O86, O111, O114, O119, O125, O126, O127, O128, O142, and O158 and is therefore less studied. Nevertheless, this aEPEC serotype has lately been recognized as an important emerging pathogen more frequently isolated from human patients with diarrhea than the typical EPEC (45). Furthermore, aEPEC serotypes have previously been reported to be common in animals such as cattle (46) and dogs (47). In a survey aimed at isolating EPEC from cattle farms and abattoirs in Ireland, 140 strains were isolated from 2,700 samples, including feces from cattle on farms, carcasses, hides, and soil. All the strains belonged to aEPEC, covering nine serotypes: O145, O2, O26, O25, O29, O98, O103, O15, and O108. Among them, O98 strains comprised 6.4% and originated mainly from fecal samples (46). In a similar survey in dogs in Brazil, the EPEC and aEPEC strains were divided into 23 serotypes (21 aEPEC and 2 EPEC serotypes) that were isolated from 13% of dogs with diarrhea and 8.3% without diarrhea. The O98 strain was also one of the aEPEC strains identified (47). In addition, O98 was reported to have been isolated from pigs with gastrointestinal disease (48) and has been isolated from humans with diarrhea (49).

In our study, given that the samples of diarrhea were negative for other common diarrhea-associated pathogens such as *Salmonella, Shigella, Campylobacter, Yersinia*, and helminths, it is likely that the aEPEC strains were the cause of diarrhea in these animals. The lack of aEPEC isolates from the samples from the other two monkeys with diarrhea could be related to reduced bacterial shedding in fecal samples or to the low temperature, which has been previously shown to decrease the number of *E. coli* when stored at −20°C for a few days (50). However, the true reason is unknown. In addition, it is necessary to identify the virus and nutrition as the cause of diarrhea in the future to access the true pathogenic factor.

Fortunately, both the EPEC isolates were sensitive to the 16 antibiotics tested. It indicates that these drugs might be used when the monkeys require some medication. This drug susceptibility may be associated with the fact that the monkeys have lived in the mountains for generations, do not receive antibiotic treatment, and are segregated from domestic animal species that may have been administered antibiotics when they are sick. Meanwhile, antibiotic-resistant genes cannot be spread from the water, soil, or other contaminants in the environment to the monkeys.

Regarding the possible source of the aEPEC strains, it is possible that they were carried by clinically healthy monkeys and later caused diarrhea as an opportunistic pathogen, as reported previously by other investigators (51). In fact, aEPEC strains could be isolated from 3.9% of normal feces from cattle (46). In this study, the overall positive rate in feces was 2.6% (2/76) (95% CI, 0.3–9.2%), which is close to the cattle carrier rate.

Despite the very limited number of samples with O98 in this study, and the fact that the pathogenicity in this monkey species remains to be determined, our finding is of importance because it is the first report of aEPEC O98 in this species of monkey. Additional studies should be performed by increasing the number of sampled monkeys and extending the sampling time frame to assess whether aEPEC O98 is a common pathogen in this species of monkey, and even other species in the wild, to evaluate fully the ecological risk of aEPEC O98.

In conclusion, this is the first study to report the presence of aEPEC O98 strains in golden snub-nosed monkeys with diarrhea. The results could be of significance in protecting this rare primate species, owing to the potential pathogenicity of these aEPEC O98 strains.

## Ethics Statement

This study was carried out in accordance with the recommendations of Hubei Regulations for the Administration of Affairs Concerning Experimental Animals (2005), Ethical Committee for Experimental Animals of Huazhong Agricultural University. The protocol was approved by the Ethical Committee for Experimental Animals of Huazhong Agricultural University (Permit Number: SYX–K(ER)2010-0029).

## Author Contributions

MQ and QW performed sample collection, performed tests, and drafted the manuscript. ST and GZ contributed to parts of the sample collection. AG and CH conceived and designed the study and corrected the manuscript. WY, YZ, XL, and JC coordinated the field work. HC and YC performed some of the experiments. RD analyzed the data. SP revised the manuscript. All authors read and approved the final manuscript.

## Conflict of Interest Statement

The authors declare that the research was conducted in the absence of any commercial or financial relationships that could be construed as a potential conflict of interest. The reviewer VN and handling Editor declared their shared affiliation.

## Footnotes

^1^http://www.iucnredlist.org.

^2^http://epitools.ausvet.com.au/content.php?page=home.
