# Supplementary material for: Identification of Atypical Enteropathogenic Escherichia coli O98 from Golden Snub-Nosed Monkeys with Diarrhea in China
Source: Front Vet Sci. 2017 Dec 13;4:217. doi: 10.3389/fvets.2017.00217 (PMC5733351; doi:10.3389/fvets.2017.00217)
Supplement: Supplementary file 1 [file table_1.doc]

Supplementary Materials

**Identification of atypical enteropathogenic Escherichia coli O98 from golden snub-nosed monkeys with diarrhea in China**

Mingpu Qi^#^, Qiankun Wang^#^, ShengtaoTong, Gang Zhao, Changmin Hu*, Xiang Li, Mingyao Liao, WanjiYang, Yingyu Chen, Sara Platto, Robertson Ian Duncan, Jianguo Chen, Huanchun Chen, Aizhen Guo*

^#^ These authors have contributed equally to this work.

***Correspondence:** Aizhen Guo: [aizhen@mail.hzau.edu.cn](mailto:aizhen@mail.hzau.edu.cn); Changmin Hu: hcm@mail.hzau.edu.cn.

# Supplementary Figures and Tables

## Supplementary Tables

**Table S1 Mouse LD_50_ test for aEPEC strains from golden snub-nosed monkeys**

| Strains | Dose  (CFU in 200μl) | No. dead/ tested | Total no of dead/tested | Dead rates  % | LD50  (CFU in 200μl) |
| --- | --- | --- | --- | --- | --- |
| No 1 from the monkey YY2  No 2 from the monkey JJ  PBS Control | 7.40×10^8^  3.68×10^8^  1.85×10^8^  9.25×10^7^  4.66×10^7^  2.32×10^7^  4.72×10^8^  2.40×10^8^  1.18×10^8^  5.93×10^7^  2.95×10^7^  1.48×10^7^  200μl | 6/6  5/6  3/6  3/6  1/6  0/6  6/6  6/6  5/6  4/6  1/6  0/6  0/6 | 18/36  12/30  7/24  4/18  1/12  0/6  21/36  15/30  9/24  5/18  1/12  0/6  0/6 | 50  40  29  22  8  0  58  50  38  28  8  0  0 | 7.40×10^8^  2.40×10^8^  / |
